# Magnetic graphene oxide-ultrathin nickel–organic framework composite for the extraction and determination of epoxiconazole in food samples†

**DOI:** 10.1039/d0ra08650a

**Published:** 2020-12-18

**Authors:** Songqing Chen, Suyu Wan, Qingchun Lan, Yan Zheng, Xiashi Zhu

**Affiliations:** College of Chemistry and Chemical Engineering, Yangzhou University Yangzhou 225002 P. R. China xszhu@yzu.edu.cn zhuxiashi@sina.com; College of Guangling, Yangzhou University Yangzhou 225002 P. R. China

## Abstract

In this work, a magnetic graphene oxide-ultrathin metal–organic framework composite (Fe_3_O_4_@SiO_2_-GO-Ni-MOF) was synthesized for the first time. Employing Fe_3_O_4_@SiO_2_-GO-Ni-MOF composite as extractant, a novel method for the separation and analysis of the pesticide epoxiconazole was established with the assistance of high performance liquid chromatography (HPLC). The adsorption mechanisms were studied including by adsorption kinetics, thermodynamic parameters and adsorption isotherms. The experimental results showed that this method was convenient, operable, effective and practical for the extraction and determination of epoxiconazole in real samples.

## Abstract 2

Schematic illustration for the MSPE procedures.

## Graphical abstract

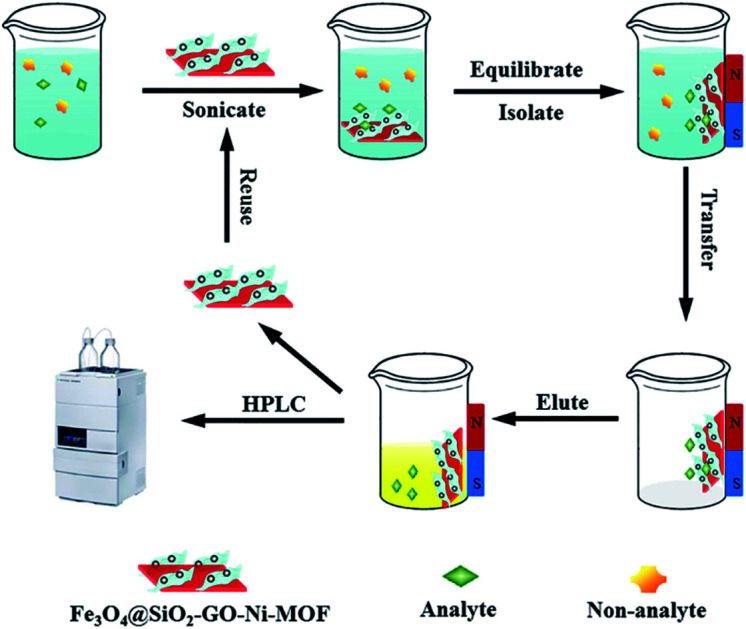

## Introduction

As a novel two-dimensional material, graphene oxide (GO)^1^ has attracted the attention of plenty of researchers because of its advantages of mechanical and chemical stability, unique electrical properties and ultrahigh surface area. Furthermore, it contains a large number of oxygen-containing functional groups, for example, carbonyl, hydroxyl and carboxyl, which guarantee the chemical activity of the materials. However, what cannot be ignored is that it is difficult to separate GO from the solutions due to its good dispersion in aqueous solutions, which limits the application of GO to a large extent. Hence, it is necessary to modify GO to ensure better properties. Fortunately, it has been confirmed that when GO is endowed with magnetism by combining magnetic materials, GO can be separated well and quickly by using appropriate additional magnetic field. Therefore, the magnetic graphene oxide (MGO) materials and^2,3^ has become as the potential adsorbents due to large adsorption area and good separation performance, indicating that the material has good.

In order to improve the selectivity and adsorption capacity of adsorption materials in larger degree, further functionalization of MGO is necessary with other kinds of materials, such as polymer,^4,5^ β-cyclodextrin,^6^ amino acid,^7^ ionic liquids,^8^ DNA,^9^ carbon nanotube^10^ and metal–organic frameworks (MOF).^11^ As a new kind of ultrathin two-dimensional nanosheets, ultra-thin MOF^12^ is attracting an increasing attention because of their large surface areas, unique spatial structure outstanding electronic properties, remarkable mechanical strength, *etc.* Ultra-thin MOF materials have been extensively studied for energy storage,^13^ catalysis,^14^ sensing,^15^ separation,^16,17^ and so on. So far, there is no report on the preparation of the nanocomposite of magnetic graphene oxide and ultrathin two-dimensional MOFs.

Currently, intense attention is focused on rising serious environmental problems and pesticide residues^18,19^ in food is one of the hot issues. Epoxiconazole^20^ (the structure showed in Fig. S1†) is a kind of triazole pesticide^21,22^ used for sterilization. It has the characteristics of strong absorption, long lasting period, good efficacy and wide application. In recent years, triazole fungicides have caused great harm to the ecological environment due to overuse and misuse. In China, the national standard stipulates the maximum residue limit (MRL) of epoxiconazole in vegetables and fruits. Such as, the MRL of epoxiconazole in apples is less than 0.5 mg kg^−1^.^23^ Therefore, it is of great significance to develop a simple, rapid and accurate analytical method for the determination of epoxiconazole. However, due to the low concentration and the complex matrix in real samples, epoxiconazole is necessary to be separated or enriched. The commonly used separation and enrichment methods include dispersive liquid–liquid microextraction,^24^ rotating disk sorptive extraction^25^ and magnetic solid-phase extraction (MSPE).^26^ Compared with traditional solid phase extraction, magnetic solid-phase extraction^27^ can avoid time-consuming centrifugal steps and the use of organic solvents, and can separate target analytes in crude solution. Magnetic adsorbent plays a key role in the MSPE because of its large specific surface area, short equilibrium time and high adsorption efficiency. At present, magnetic amino modified multiwalled carbon nanotubes,^26^ magnetic partially carbonized cellulose nanocrystals,^28^ carbon nanosphere@Fe_3_O_4_ (ref. 29) and ionic liquid-based magnetic carbon nanotubes^30^ have been used as adsorbents for magnetic solid phase extraction of epoxiconazole. However, no studies have been reported on the magnetic solid phase extraction of epoxiconazole using MGO or MGO composite.

In this paper, a novel kind of magnetic graphene oxide-ultrathin metal–organic frameworks nanocomposite (Fe_3_O_4_@GO-Ni-MOF) was prepared for the first time, and further applied to the separation and analysis of epoxiconazole in food combining with high performance liquid chromatography (HPLC) (showing in Fig. S2†). The nanocomposite was synthesized by *in situ* method, and then characterized by Scanning electron microscopy (SEM) and transmission electron microscopy (TEM) and Fourier transform infrared spectra (FT-IR). The Fe_3_O_4_@GO-Ni-MOF materials presented special ultrathin two-dimensional structure, which enlarges the surface area of the adsorbents and further enhances the properties of the materials. Moreover, the proposed strategy can been applied to the separation and analysis of epoxiconazole in real samples with satisfactory results.

## Experimental

### Magnetic solid-phase extraction procedures

**Fig. 1:**
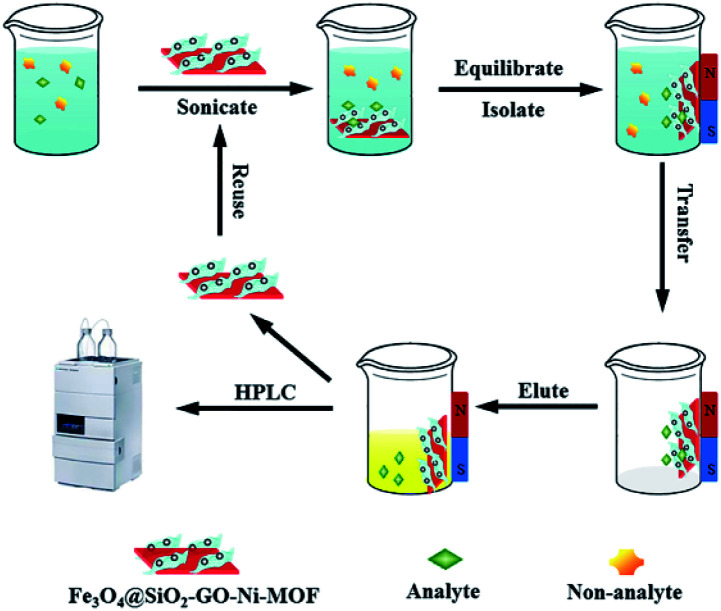
Schematic illustration for the MSPE procedure.

The schematic illustration for the MSPE procedures were shown in Fig. 1. Proper amount of epoxiconazole solution was added to the centrifugal tube, as well as 5.0 mL buffer solution of pH 7.0, then the volume was fixed to 30.0 mL. Fe_3_O_4_@SiO_2_-GO-Ni-MOF composites were accurately weighed at 5.0 mg and oscillated for 14 min at 35 °C. The supernatant was poured out, 2.0 mL acetone was added to the centrifugal tube and vibrated for 10 min, and then the supernatant was separated by magnet and determined by Agilent 1120 HPLC.

## Results and discussion

### Characterization of the Fe_3_O_4_@SiO_2_-GO-Ni-MOF

**Fig. 2:**
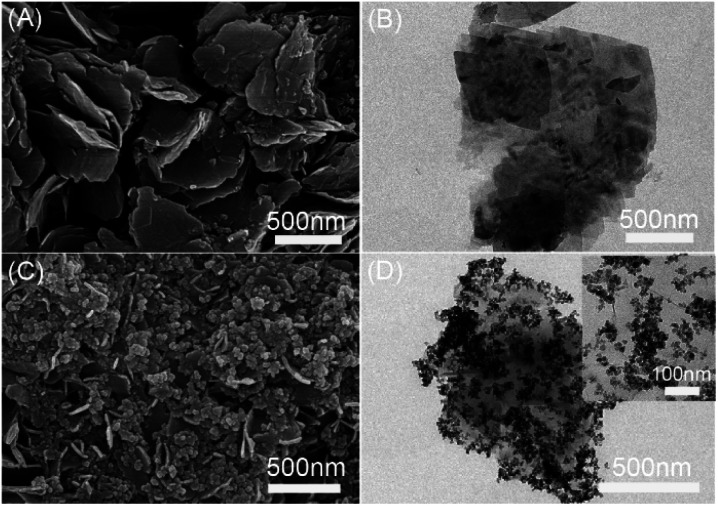
SEM of (A) Ni-MOF and (C) TEM of Fe_3_O_4_@SiO_2_-GO-Ni-MOF, TEM of (B) Ni-MOF and (D) Fe_3_O_4_@SiO_2_-GO-Ni-MOF.

Scanning electron microscopy (SEM) and transmission electron microscopy (TEM) were employed to reveal the morphology of the surface. The results are shown in Fig. 2. From Fig. 2(A) and (B), it can be seen that the Ni-MOF was a two-dimensional nano sheet material. Compared with Fig. 2(C) and (D), it can be seen that there are clusters of spherical nanoparticles (Fe_3_O_4_@SiO_2_) on Ni-MOF sheets. Furthermore, it can be seen by enlarging the magnification Fe_3_O_4_@SiO_2_ nanoparticles are attached to graphene oxide sheet, which indicates that Fe_3_O_4_@SiO_2_-GO-Ni-MOF was been successfully prepared.

**Fig. 3:**
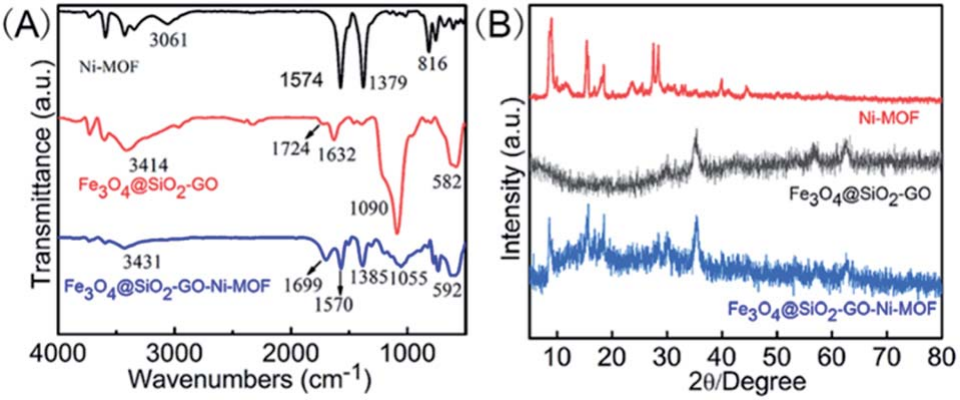
(A) FTIR spectra and (B) XRD patterns of Ni-MOF, Fe_3_O_4_@SiO_2_-GO and Fe_3_O_4_@SiO_2_-GO-Ni-MOF.

As shown in Fig. 3(A), Fourier transform infrared spectra (FTIR) of Fe_3_O_4_@SiO_2_-GO, Ni-MOF and Fe_3_O_4_@SiO_2_-GO-Ni-MOF were recorded within the wavenumber range of 4000–500 cm^−1^, which also proved the successful synthesis of the materials. In the curve of Ni-MOF, the peak at 3061 cm^−1^ was corresponded to the stretching vibration of O–H, the peaks of 1574 cm^−1^, 1379 cm^−1^ were caused by asymmetric and symmetric stretching vibration of –COO^−^, and the peak at 816 cm^−1^ can be explained by *para* disubstitution of benzene ring. In the infrared spectrum of Fe_3_O_4_@SiO_2_-GO, the bands at around 3414 cm^−1^, 1724 cm^−1^, 1632 cm^−1^, 1090 cm^−1^ and 582 cm^−1^ symbolized the stretching vibration peak of O–H and C=C, the bending vibration of water molecule, the antisymmetric stretching vibration peak of Si–O–Si, as well as the stretching vibration peak of Fe–O, respectively. The characteristic absorption peaks of Ni-MOF and Fe_3_O_4_@SiO_2_-GO were well reflected in the infrared spectrum of Fe_3_O_4_@SiO_2_-GO-Ni-MOF, indicating that the nanocomposite was successfully synthesized.

As shown in Fig. 3(B), in order to further characterize the crystal structure of Fe_3_O_4_@SiO_2_-GO-Ni-MOF, the X-ray diffraction (XRD) diagram is measured. It could be seen from the figure that the nanocomplex had not only the characteristic peaks of Ni-MOF (2*θ* = 9.0°, 15.4°, 18.5°, 27.6°, 28.4°), but also Fe_3_O_4_@SiO_2_-GO (2*θ* = 35.4°, 56.6° and 62.4°), indicating that the synthesis of Fe_3_O_4_@SiO_2_-GO-Ni-MOF composite was successful.

The magnetic properties of nanocomposite was studied by VSM and the results were showed in Fig. S3.† As can be seen, the saturation magnetization intensity of Fe_3_O_4_@SiO_2_, Fe_3_O_4_@SiO_2_-GO and Fe_3_O_4_@SiO_2_-GO-Ni-MOF were 66.2 emu g^−1^, 34.8 emu g^−1^ and 23.8 emu g^−1^, respectively. It was obvious that the saturation magnetization intensity of the composite was obviously reduced, which indicated that the composite has been successfully synthesized.

### Optimization of extraction and elution conditions

The factors that may affect the extraction efficiency of epoxiconazole were studied in Fig. S4 and S5.† The results showed that the optimum extraction conditions were Fe_3_O_4_@SiO_2_-GO-Ni-MOF (5.0 mg) and pH (7.0–14.0), extraction time (14.0 min), extraction temperature (35 °C), the volume of the sample (30.0 mL) and the optimum elution conditions were acetone (2.0 mL), elution time (10.0 min), elution temperature (30 °C).

### Adsorption mechanism

In order to explore the adsorption mechanism of MGO-Ni-MOF composite on epoxiconazole, the enthalpy (Δ*H*), Gibbs free energy (Δ*G*) and entropy (Δ*S*) of the adsorption process were calculated based on eqn (1) and (2).

1 ln *Q*_e_/*C*_e_ = −(Δ*H*)/*RT*(1/*T*) + (Δ*S*)/*R*

2 Δ*G* = Δ*H* − *T*Δ*S*

**Fig. 4:**
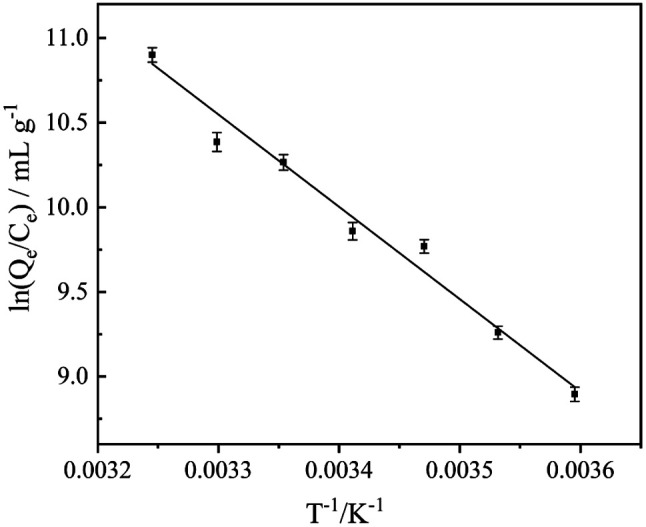
Thermodynamic parameters.

At 5.0–35.0 °C, ln(*Q*_e_/*C*_e_) was plotted with 1/T according to formula (1), as shown in Fig. 4. Combining with formula (2), the adsorption thermodynamic parameters Δ*G*, Δ*H*, Δ*S* are obtained (Table 1): thermodynamic parameters Δ*G* < 0 illustrates a spontaneous adsorption process; Δ*H* > 0 suggests an endothermic adsorption process and Δ*H* = 44.6 kJ mol^−1^ indicates the reaction is chemical adsorption; Δ*S* < 0 shows that the adsorption process increases the degree of freedom of the system.

**Table 1:** The results of thermodynamics

*T* (K)	Δ*G* (kJ mol^−1^)	Δ*H* (kJ mol^−1^)	Δ*S* (J mol^−1^ K^−1^)	*R* ^2^
278.15	−20.67	44.64	234.8	0.9781
283.15	−21.84			
288.15	−23.02			
293.15	−24.19			
298.15	−25.36			
303.15	−26.54			
308.15	−27.71			

**Fig. 5:**
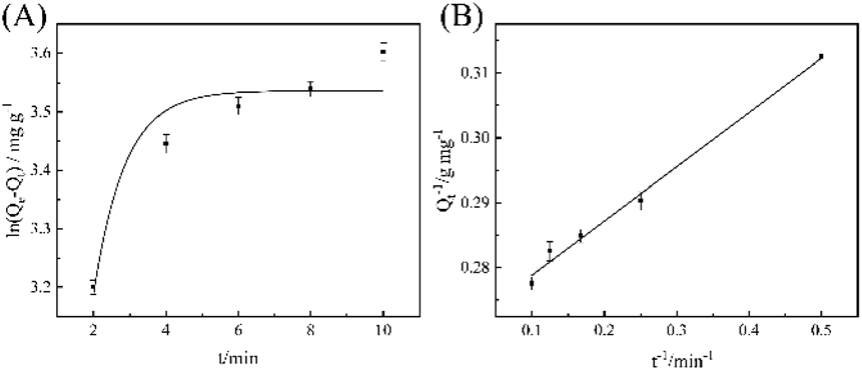
(A) Pseudo-first-order kinetics model. (B) Pseudo-second-order kinetics model.

To further study the adsorption mechanism of MGO-Ni-MOF on epoxiconazole, the experimental results were analyzed by pseudo-first-order kinetics and pseudo-second-order kinetic, which can be seen in Fig. 5. The correlation coefficients (*R*^2^) of pseudo-first-order kinetics is 0.9136, while that of pseudo-second-order kinetics is 0.9923. The results show that pseudo-second-order kinetics model can be more appropriate for describing the detecting process and this indicates that it is chemisorption, which is consistent with thermodynamic results.

The adsorption isotherm model of Fe_3_O_4_@SiO_2_-GO-Ni-MOF composite materials on the pesticide epoxiconazole was also examined by Langmuir and Freundlich adsorption isothermal formulas, which are commonly utilized to check the experimental data.

**Fig. 6:**
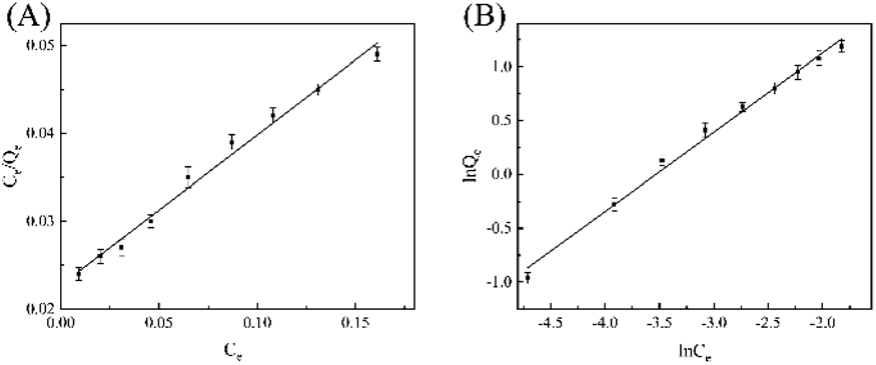
(A) Langmuir adsorption isotherm and (B) Freundlich adsorption isotherm of epoxiconazole in Fe_3_O_4_@SiO_2_-GO-Ni-MOF composite.

As results shown in Fig. 6, the linear correlation coefficients of Langmuir model and Freundlich model are 0.9892 and 0.9926, respectively. Therefore, Freundlich adsorption isotherm model is more suitable for the adsorption of epoxiconazole on MGO-Ni-MOF composites.

### Analytical performance

The analytical performance data for the epoxiconazole by the proposed method was listed in Table S1.† Under the optimum conditions, the calibration graph was linear in the range 0.01–2.00 μg g^−1^. The calibration equation is *I* (intensity) = 81.6*c* + 2.05 (μg g^−1^) with a correlation coefficient (*R*^2^) of 0.9989. The limit of detection (LOD), defined as LOD = 3*S*_B_/*m*, where *S*_B_ and *m* are standard deviation of the blank and the slope of the calibration graph, respectively, was 0.001 μg g^−1^. The limit of quantification (LOQ) was 0.004 μg g^−1^. The relative standard deviation (RSD) was 3.6% (*c* = 0.2 μg g^−1^, *n* = 3). The enhancement factor (EF), defined as the volume ratio before and after extraction, was 15.

The reusability of the adsorbent was explored in Fig. S5† and it could be used 6 times.

The possible interfering substances in real samples were studied in Table S1† and the results indicated that it has good resistance to the interference of external substances.

### Analysis of real samples

To evaluate to the applicability of the proposed method, it was employed in the analysis of epoxiconazole in fruits and vegetables samples, such as cabbage, apple, pear, cucumber, tomato and celery purchased from local market. The results are presented in Table 2. According to the table, it can be seen that the recoveries of the samples are in the range of 91.0% to 112.4% and there is no epoxiconazole in the six samples. The results meet the standards of GB 2763-2019.

**Table 2:** Analysis of fruits and vegetables samples

Samples	Added (μg g^−1^)	Found (μg g^−1^)	Recovery (%) (*n* = 3)
Cabbage	0	ND^a^	—
	0.100	0.107	107.0
	0.500	0.523	104.6
Apple	0	ND	—
	0.100	0.105	105.0
	0.500	0.562	112.4
Pear	0.00	ND	—
	0.100	0.091	91.0
	0.500	0.486	97.2
Tomato	0	ND^a^	—
	0.100	0.095	95.0
	0.500	0.486	97.2
Celery	0	ND	—
	0.100	0.094	94.0
	0.500	0.462	92.4
Cucumber	0	ND	—
	0.100	0.102	102.0
	0.500	0.532	106.4

a ND, not detected.

## Conclusions

To conclude, Fe_3_O_4_@SiO_2_-GO-Ni-MOF composite was successfully synthesized and characterized by various means. The composite integrates the advantages of each component and give better synergistic effect to the composite material. The Fe_3_O_4_@SiO_2_-GO-Ni-MOF materials were used to separate and analyze epoxiconazole and showed excellent extraction performance than each single component. The important factors of this experiment which affect the extraction rate and elution were optimized. Furthermore, this method was successfully used to detect the situation of epoxiconazole pesticide in some fruits and vegetables in vegetable market. This Fe_3_O_4_@SiO_2_-GO-Ni-MOF based method is feasible, practical and will play a great role in the extraction of pesticide epoxiconazole in the near future. More complex samples may need to be combined with MS.

## Conflicts of interest

There are no conflicts to declare.

## Supplementary Material

RA-010-D0RA08650A-s001
