# Supplementary material for: Magnetic graphene oxide-ultrathin nickel–organic framework composite for the extraction and determination of epoxiconazole in food samples
Source: RSC Adv. 2020 Dec 18;10(73):44793–7. doi: 10.1039/d0ra08650a (PMC9058641; doi:10.1039/d0ra08650a)
Supplement: RA-010-D0RA08650A-s001 [file RA-010-D0RA08650A-s001.pdf]

Electronic Supplementary Material (ESI) for RSC Advances.
This journal is © The Royal Society of Chemistry 2020

Electronic Supplementary Information (ESI) for RSC Advances

Magnetic graphene oxide-ultrathin nickel-organic frameworks composite for the extraction and determination of epoxiconazole in food samples

Songqing Chen, Suyu Wan, Qingchun Lan, Yan Zheng, Xiashi Zhu*

1 *College of Chemistry and Chemical Engineering, Yangzhou University, Yangzhou 225002, P.R. China*

2 College of Guangling, Yangzhou University, Yangzhou University, Yangzhou 225002, PR China

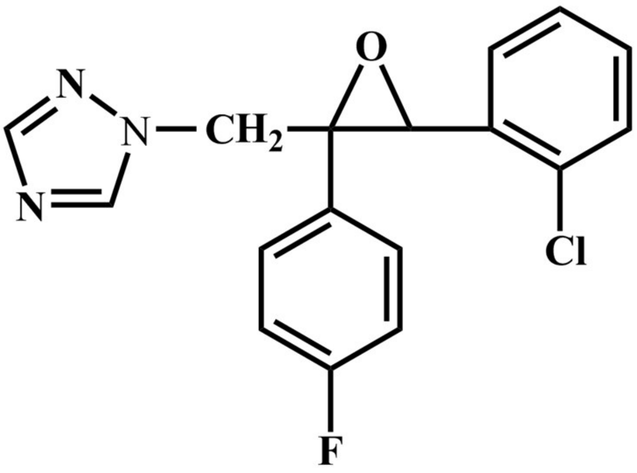

Fig. S1 The structure of epoxiconazole

Experimental

Materials and Reagents. $(NH_4)_2Fe(SO_4)_2 \cdot 6H_2O$, $(NH_4)_2Fe(SO_4)_2 \cdot 12H_2O$, anhydrous ethanol, tetraethyl orthosilicate, isopropanol, acetone, acetonitrile, n-hexane, N-hydroxysuccinimide (NHS), 1-ethyl-(3-dimethylaminopropyl) carbamide acetate (EDC), terephthalic acid (PTA), sodium hydroxide, sodium chloride, nickel nitrate hexahydrate, N, N-dimethyl Methyl formamide (DMF), (3-aminopropyl) triethoxysilane (APTES), methanol, anhydrous ferric chloride, polyethylene glycol (PEG) were purchased from the Sinopharm Chemical Reagent Limited Corporation (Shanghai, China), multilayer graphene oxide was purchased from Suzhou Tanfeng graphene Tech Co., Ltd. (Suzhou, China). Except for the use of ultrapure water in high performance liquid chromatography (HPLC), all the experimental water was

distilled water.

Apparatus. DK - S22 electrothermal constant temperature water-bath (Changzhou guohua electric appliance co., LTD.), KQ-50E ultrasonic cleaning machine (Kunshan ultrasonic instrument co., LTD), vacuum drying oven (Shanghai yiheng scientific instrument co., LTD. (Shanghai), Sartoeius electronic balance (STOLUS scientific instruments co., LTD.), DGG-9030-B electrothermal constant temperature drying box (Shanghai senxin experimental instrument co., LTD.), SH-C thermostatic oscillator (Changzhou guohua electric appliance co., LTD.), TG16-WS table high speed centrifuge, electric stirrer (Changzhou guohua electric appliance co., LTD.), Mettler Toledo pH meter (Shanghai jingke leici instrument co., LTD), Tensor27 Fourier infrared spectrometer (Germany Bruker company), OSB-2200 rotary evaporation instrument (EYELA Tokyo physical and chemical equipment co., LTD), TDL80-2B centrifuge (Shanghai anting scientific instrument factory), Florisil silica solid-phase extraction column (yide biological technology co., LTD.), Agilent Technologies 1120 Infinity high performance liquid chromatograph with UV detector. (Agilent Technologies co., LTD.).

Prepare of Fe_3O_4@SiO_2-GO-Ni-MOF. Fe_3O_4 NPs were synthesised via the conventional co-precipitation preparation.[S1] Firstly, 3.24 g of $(NH_4)_2Fe(SO_4)_2 \cdot 6H_2O$ and 3.92 g $(NH_4)_2Fe(SO_4)_2 \cdot 12H_2O$ were dissolved in 100 mL deionized water, Next, 30 mL 10% polyethylene glycol and 10 mL ammonium hydroxide were added into the above solution, which was then stirred at 80 ^oC for 60 min. Finally, a magnet was placed at the bottom of the beaker, the solution was dumped, and the product of Fe_3O_4 were collected. Then, the product were obtained after being washed with deionized water, followed by being dried at 60 ^oC for 12 h.

GO was synthesized via modified Hemmers methods. [S2] Firstly, 2.0 g of graphite and 1.0 g of $NaNO_3$ were mixed into 46 mL of 98% H_2SO_4, and stirred for 1 h at 0 ^oC. Afterwards, 6.0 g of $KMnO_4$ was slowly added into the mixture and continued to keep reaction temperature at 0 ^oC for 2 h. Then, the mixture was taken away from the ice bath, and stirred for 30 min at 38 ^oC. Next, 160 mL deionized water was added into above mixture, and its temperature increased to 95 ^oC. After keeping a reaction temperature at 95 ^oC for 30 min, 120 mL deionized water and 30 mL 3% H_2O_2 were added to reduce the

residual permanganate and manganese dioxide. The mixture was filtered when the color changed to brilliant yellow and washed with 80 mL 5% HCl aqueous solution and deionized water. After several centrifugations the final dry GO products were obtained in vacuum at 50 ^{o}C.

Preparation of Fe_3O_4@SiO_2-GO was according to the literatures with some modifications. [S3,4] First, 0.01 mol Fe_3O_4 was added in 250 mL three-port flasks, then 340.0 mL ethanol was also added twice, followed with 60.0 mL distilled water and 15.0 mL ammonia water, then ultrasound for 20 minutes. At the end of ultrasound, 1.7 mL tetraethyl orthosilicate was added and stirred for 12 hours at 60 ^{o}C in water bath. After separating the magnet, the product was washed with water and ethanol, the obtained Fe_3O_4@SiO_2 was dispersed with 140.0 mL isopropanol, which was added twice, followed by 0.20 mL (3-aminopropyl) triethoxysilane (APTES). After ultrasonicing for 30 minutes under the protection of nitrogen, the materiaks were stirred for 6 hours at 70 ^{o}C in water bath. Fe_3O_4@SiO_2-NH_2 was obtained by vacuum drying after three times of distilled water washing followed by ethanol washing. To acquire final product, 0.20 g GO was added in 100 mL three-port flask, then added 50.0 mL N, N-dimethylformamide (DMF), 0.10 g N-hydroxysuccinimide (NHS) and 0.20 g 1-ethyl-(3-dimethylaminopropyl) carbonyl diamide acetate (EDC). After ultrasonicing 20 minutes to dissolve adequately, the solution pH should be adjusted to 4.0-6.0. After the intense stirring at room temperature for 2 hours, then add Fe_3O_4@SiO_2-NH_2, continue to 12 hours. After the reaction, magnet was used to separate, distilled water was washed for 3 times, and finally air dried to get Fe_3O_4@SiO_2-GO.

Prepare of Fe_3O_4@SiO_2-GO-Ni-MOF. Firstly, 0.10 g Fe_3O_4@SiO_2-GO was added in 100 mL three-port flask, followed by adding 0.166 g terephthalic acid (PTA) and 10.0 mL N, N-dimethylformamide (DMF), stirring 15 minutes.[S5] Then, the solution was added by 2.0 mL 0.40 mol L^{-1} sodium hydroxide and stirred for 10 minutes. In another small beaker, 0.096 g $Ni(NO_3)_2 \cdot 6H_2O$ and 10.0 mL N, N-dimethylformamide (DMF) were added, stirring to dissolve $Ni(NO_3)_2 \cdot 6H_2O$ completely, then they was transferred to the above solution, stirred for 30 minutes. After the reaction, the solution was poured into the reactor and reacted for 8 hours at 100 ^{o}C. Fe_3O_4@SiO_2-GO-Ni-MOF composites were prepared by air-drying after separation with magnet, washed with N, N-dimethylformamide (DMF) and absolute

ethanol three times respectively.

Sample preparation. Fruits and Vegetables: Put the cut fruits and vegetables samples into the juicer to crush. After crushing, add 10.0 g sample, 20.0 mL of acetonitrile and 2.0 g of sodium chloride into the centrifugal tube, and shake for 15 minutes to dissolve fully. After the oscillation, the centrifugal tube was put into the centrifuge for 10 minutes. The rotational speed of the centrifuge was 8000 r min^{-1}. After the separation of acetonitrile and water, the upper acetonitrile was removed, and then 20.0 mL acetonitrile was added to the centrifugal tube for secondary extraction. The centrifugal operation was repeated and the acetonitrile extract obtained by two centrifugations was rotated at 100 mL. In the rotary evaporation bottle, the extract was concentrated to dry at 50 ^oC by rotary evaporator. 2.0 mL n-hexane was added to dissolve the steam-dried extract, which was added into Florisil after its activation. Then, 5.0ml of acetone and n-hexane were mixed respectively, eluent was collected by elution column, and the eluent was concentrated to dry by rotary evaporator at 50 ^oC. 2.0 mL of ethanol was added to the evaporated eluent to dissolve it, and the dissolved solution was collected for determination .

Chromatographic Conditions. Chromatographic column: shin-pack VP-ODS C_{18} (250 mm×4.6 mm,5μm), mobile phase: methanol-water (85:15, V/V), flow rate: 0.80 mL min^{-1}, column temperature: 25℃, detection wavelength: 205 nm, injection volume: 20 μL,peak position of epoxiconazole is 6.38 min, as shown in Fig. S2.

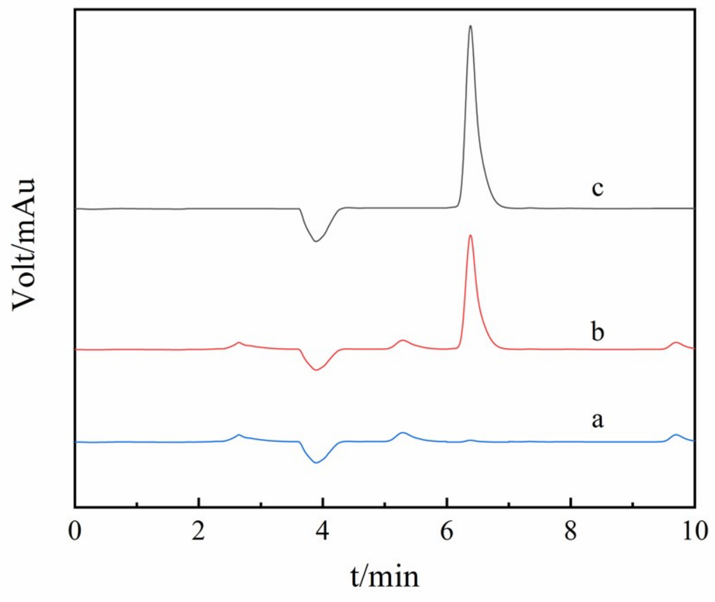

Fig. S2 Chromatograms of (a) cabbage sample, (b) the same cabbage sample spiked at 0.1 μg mL^{-1} and (c) water sample spiked at 0.2 μg mL^{-1}

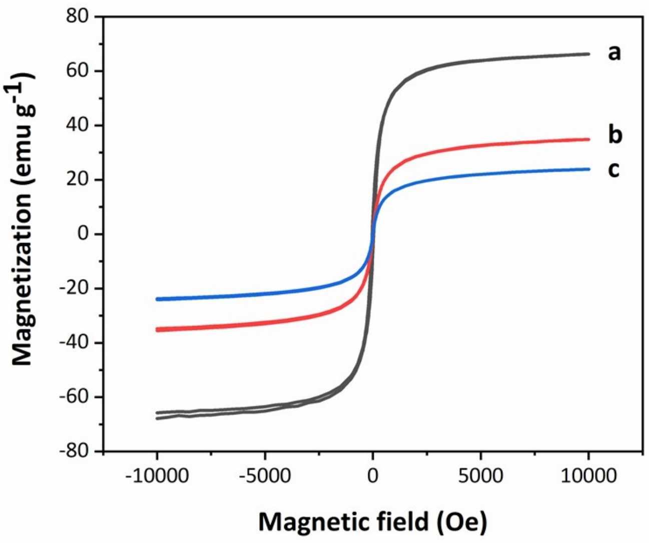

Fig. S3. Magnetization curves of (a) Fe_3O_4, (b) Fe_3O_4@SiO_2-GO, (c) Fe_3O_4@SiO_2-GO-Ni-MOF

Optimization of extraction conditions.

The type of adsorbent is critical to the extraction process，in order to select the best material for the extraction of epoxiconazole, the effects of Fe_3O_4, Fe_3O_4@SiO_2, Fe_3O_4@SiO_2-GO, Fe_3O_4@SiO_2-GO and Fe_3O_4@SiO_2-GO-Ni-MOF on the Extraction efficiencies of epoxiconazole were studied in Fig. S4 (A). As can be seen, the extraction efficiency of Fe_3O_4@SiO_2-GO-Ni-MOF is higher than the others, which may be due to the large specific surface area of GO and the good solubility of Ni-MOF to epoxiconazole. Therefore, Fe_3O_4@SiO_2-GO-Ni-MOF was chosen as the adsorbent.

In order to investigate the influence of the amount on the extraction efficiency, the dosages of the adsorbent in the range of 2.0 to 30.0 mg were studied in Fig. S4 (B). When the dosage of adsorbent reached or exceeded 5.0 mg, the extraction efficiency of epoxiconazole reached the maximum and remained basically constant. Therefore, the optimum dosage of nanocomposite is 5.0 mg.

The pH of sample solution can affect the existence form of adsorbent and analyte, which is another important factor affecting the extraction. This experiment studied the effect of pH on the extraction efficiency of epoxiconazole in the range of 3.0 to 14.0 in Fig. S4 (C). The extraction efficiency increased slowly in the range of pH 3.0-6.0, and the extraction efficiency was higher than 90% in the range of pH 7.0-14.0. The extraction efficiency of 85% is considered quantitative extraction. Epoxiconazole can be quantitatively extracted when pH of the solution was in the range of 7.0-14.0. . The pH is optimum in range 7-14.

Extraction time is an important factor affecting the extraction efficiency. Therefore, the effect of extraction time of 2min -18 min was investigated with an interval of 2 minutes. According to the results in Fig. S4 (D), when the extraction time is less than 14 minutes, the extraction efficiency increases with the time while when the shaking time is more than 14 minutes, the extraction efficiency remains basically unchanged, so the extraction time was choosen as 14 minutes.

In the extraction process, temperature also has a certain effect on the extraction efficiency. The extraction at different temperatures (0-40 ^{o}C)and the results can be seen in Fig. S4 (E). The extraction efficiency showed an upward trend below 35 ^{o}C. When the temperature was 35 ^{o}C, the extraction

efficiency remained reached the maximum. The adsorption of epoxiconazole to Fe_3O_4@SiO_2-GO –Ni-MOF is an endothermic process, and the increase of temperature is beneficial to the extraction (adsorption), so the extraction efficiency increases with the increase of temperature. But, when the temperature rises again, the adsorption decreases because of the increase in the velocity of the target analyte molecules. Therefore, the optimum extraction temperature was 35 ^{o}C.

The volume of the sample was optimized from 10ml to 50ml and the results were shown in the Fig. S4 (F), which illustrated that when the sample volume was less than 30 mL, the extraction efficiency is higher than 85%, which can be used for quantitative extraction. The volume of the sample was 30mL.

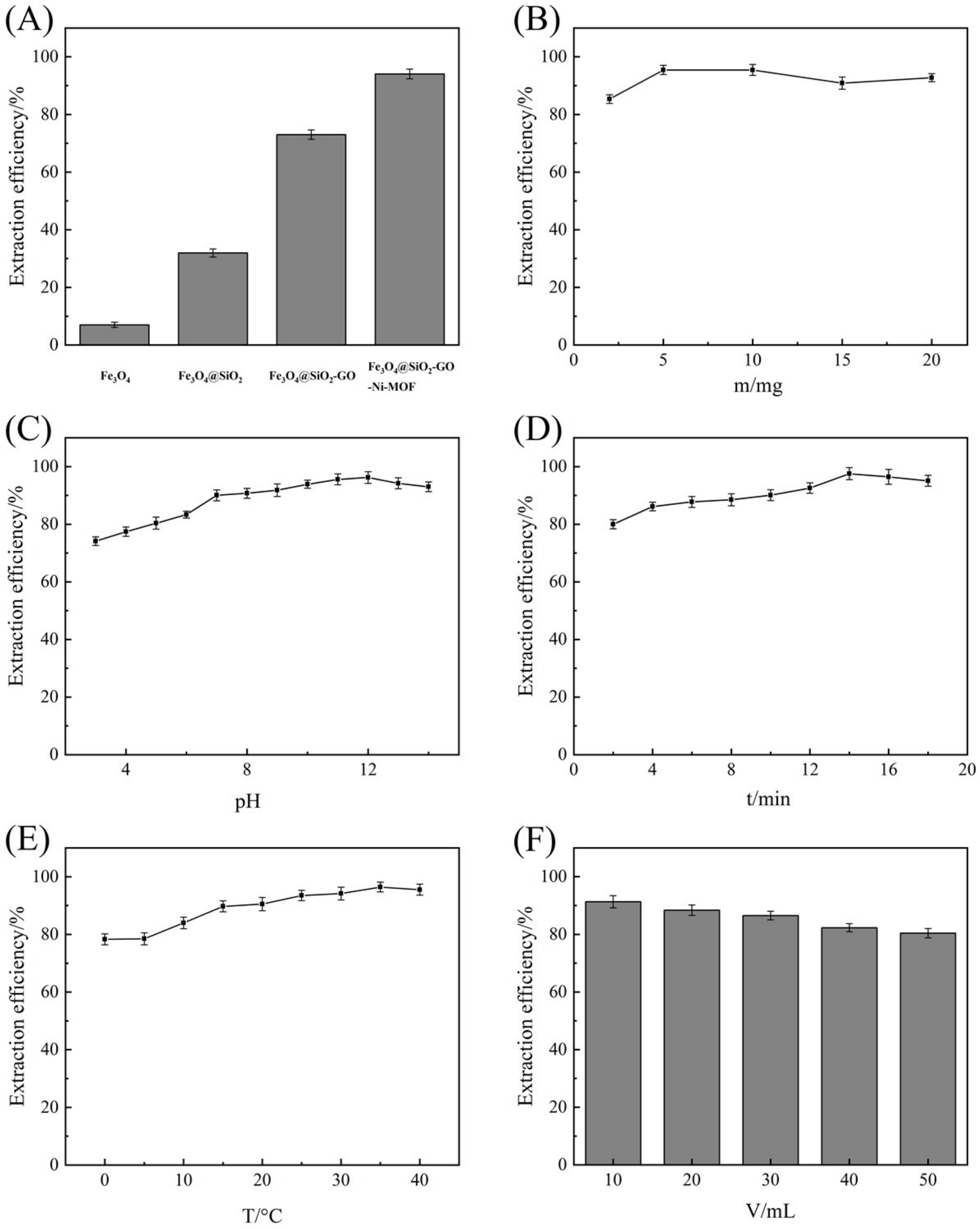

Fig. S4 Selection of adsorbent (A) and the extraction efficiency of Fe_3O_4@SiO_2-GO-Ni-MOF composite: amount of adsorbent (B), pH (C), extraction time (D), temperature (E) and volum of the sample (F).

Optimization of elution conditions. In the process of elution of epoxiconazole, many factors will affect the elution effect. Therefore, the effects of eluent types, eluent volume, elution time and elution temperature on the elution rate of epoxiconazole extracted from composite materials were studied.

In this experiment, hydrochloric acid, methanol, ethanol, acetonitrile and acetone were used to elute epoxiconazol. As shown in Fig. S5 (A), acetone was the best of all and acetone was selected as eluent.

Meanwhile, the volum of eluent (Fig. S5 (B)) was investigated in the range from 1.0 mL to 6.0 mL and it was found that when the volum was 2.0 mL, the elution effect was best and and remained unchanged with the with the increase of eluent volume. Thus, the volume of eluent was 2.0 mL.

The elution time has a certain influence on the elution effect of epoxiconazole and it was studied in the range of 5-25min. The The results were presented in Fig. S5 (C), which shown that when the elution time is less than 10.0 minutes, the elution effect increases with time. When the elution time is more than 10.0 minutes, the elution rate hardly changes, so the elution time was 10.0 min.

Moreover, the elution temperature from 0-40 ℃. were optimized. The results (Fig. S5 (D)) showed that the elution effect increased with temperature when it was less than 30 ^{o}C, and remained unchanged when the elution temperature was higher than 30 ^{o}C, so the selected elution temperature was 30 ^{o}C.

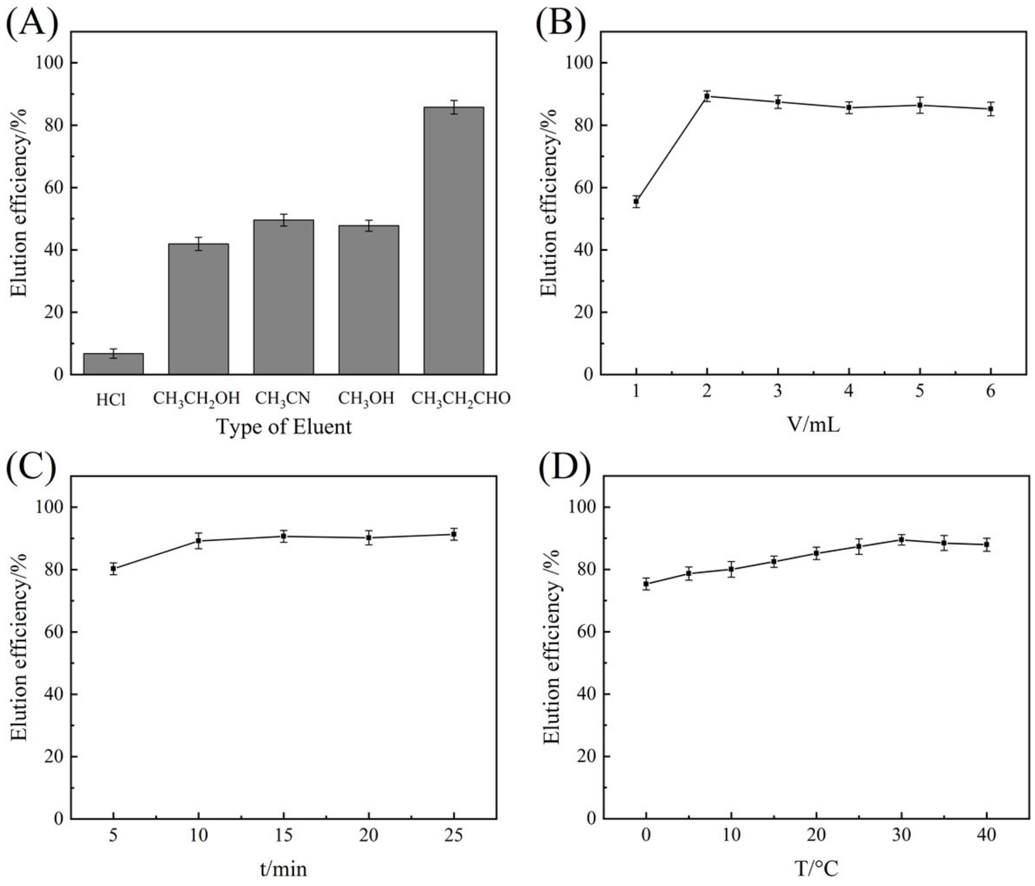

Fig. S5 The elution efficiency of Fe_3O_4@SiO_2-GO-Ni-MOF composite: eluents(A), amount of eluents (B), elution time (C) and temperature (D).

Reusability of the adsorbent. Considering the maximization of material utilization, the reusability of the adsorbent was explored in this experiment. As shown in Fig. S5, the nanocomposites can be reused for six times with the extraction efficiency more than 85%.

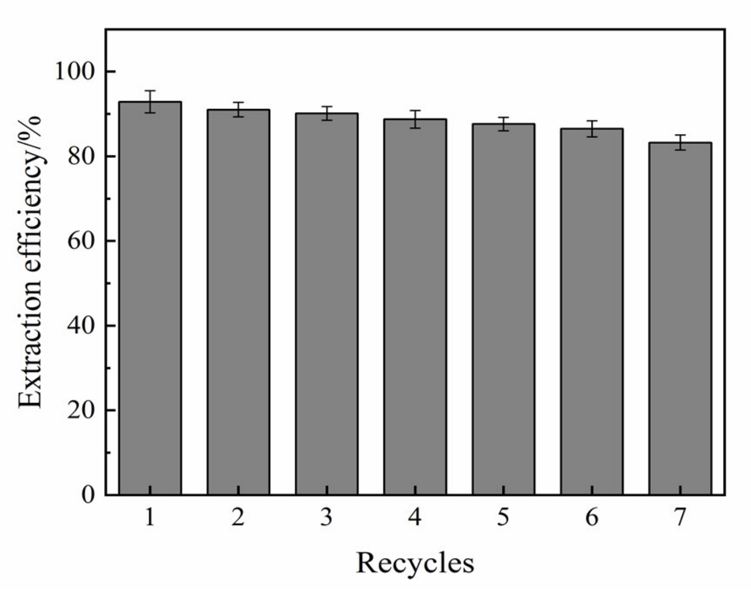

Fig. S5. Reusability of the adsorbent.

Table S1 The analytical performance data for the epoxiconazole by the proposed method

Linear range (μg mL^{-1})	R^2	LOD (μg mL^{-1})	LOQ (μg mL^{-1})	RSD (%) (n = 3)	EF
0.01-2.0	0.9989	0.001	0.004	3.6	15

Influence of interfering substances. The effect of some common metal ions and six kinds of pesticides (validamycin A, prothioconazole, tebuconazole, azoxystrobin, kasugamycin and fipronil) on the determination of epoxiconazole by the proposed method was investigated. A substance was considered as interfering when it caused a variation in the determination of the sample greater than ±5%. The results are shown in Table S2.

Table S2 Effect of interfering substances on extraction efficiency

Tested substances	Tested substances to analyte ratio(w/w)
Ca^{2+}	500
Na^{+}	500
Mg^{2+}	500
Ni^{2+}	500
Al^{3+}	500
Pb^{2+}	500
validamycin A	10
prothioconazole	5
tebuconazole	5
azoxystrobin	5
kasugamycin	5
fipronil	5

Table S3 summarizes the features of the proposed method with other methods for the determination of epoxiconazole based on HPLC. It shows that the limit of detection of this method is lower than that of other methods. Meanwhile, more samples can be determined by the proposed method with good recoveries.

Table S3 Comparison of the proposed method with other methods for the determination of epoxiconazole based on HPLC

Method	Extractant	LOD (ng mL^{-1})	LOQ (ng mL^{-1})	Linear range (μg mL^{-1})	Recovery (%)	Sample	Ref.
DLLME-HPLC	1-dodecano	5.1	16.9	0.05–5.0	79.6-104.4	Tap, reservoir, and river water	S6
DLLME-HPLC	[C_6MIM][PF_6]	10.0	30.0	0.03-15.0	76.4-100.7	Peach, apple, orange juices	S7
DLLME-HPLC	[C_8MIM][PF_6]	6.5	16.4	0.03–10.0	83.4-119.1	Pear, apple, grapefruit	S8
MSPE-HPLC	Fe_3O_4@SiO_2-GO-Ni-MOF	1.0	4.0	0.01-2.0	91.0 - 112.4	Cabbage, apple, pear, cucumber, tomato and celery	this work

References

S1 W. S. Hummers and R. E. Offeman, *J. Am. Chem. Soc.*, 1958, **80**, 1339.

S2 W. Wang, Y. Li, Q. Wu, C. Wang, X. Zang and Z. Wang, *Anal. Methods*, 2012, **4**, 766–772.

S3 Q. Liu, J. Shi, M. Cheng, G. Li, D. Cao and G. Jiang, *Chem. Commun.*, 2012, **48**, 1874–1876.

S4 Q. Liu, J. Shi, J. Sun, T. Wang, L. Zeng and G. Jiang, *Angew. Chemie - Int. Ed.*, 2011, **50**, 5913–5917.

S5 S. Zheng, B. Li, Y. Tang, Q. Li, H. Xue and H. Pang, *Nanoscale*, 2018, **10**, 13270–13276.

S6 X. Jing, L. Yang, W. Zhao, F. Wang, Z. Chen, L. Ma, L. Jia and X. Wang, *J. Chromatogr. A*, 2019, **1597**, 46–53.

S7 Y. Zhang, Y. Zhang, Q. Zhao, W. Chen and B. Jiao, *Food Anal. Methods*, 2016, **9**, 596–604.

S8 Y. Zhang, Y. Zhang, J. Nie, B. Jiao and Q. Zhao, *Food Anal. Methods*, 2016, **9**, 3509–3519.
